# Chromatographic Assessment of Organic Compounds Using Carbon Nanotubes: The Relationship between Affinity and Dispersibility

**DOI:** 10.3390/nano14100824

**Published:** 2024-05-08

**Authors:** Taiyo Shimizu, Ryoichi Kishi, Atsushi Hirano, Ken Kokubo, Kenji Hata

**Affiliations:** 1 Nano Carbon Device Research Center, National Institute of Advanced Industrial Science and Technology (AIST), AIST Tsukuba Central 5, 1-1-1 Higashi, Tsukuba 305-8565, Ibaraki, Japan; shimizu-t@aist.go.jp (T.S.);; 2 Nanomaterials Research Institute, National Institute of Advanced Industrial Science and Technology (AIST), AIST Tsukuba Central 5, 1-1-1 Higashi, Tsukuba 305-8565, Ibaraki, Japan

**Keywords:** carbon nanotube, chromatography, retention time, Hansen solubility parameter, dispersibility

## Abstract

The affinity between carbon nanotubes (CNTs) and organic compounds is of substantial importance since it strongly relates to the dispersibility of CNTs in those compounds. Several affinity evaluation methods have been developed so far, and the concept of the Hansen solubility parameter is a representative method widely used in the field of nanocarbon materials. Here, we demonstrate that CNT-loaded silica columns can effectively assess the affinity of organic compounds for CNT surface by exploiting the chromatographic retention time as a criterion. Obtained trends of the affinity of organic compounds for CNT were compared to those based on Hansen solubility parameter distance values. Most organic compounds showed similar trends, but one exceptional compound was observed. Simple CNT dispersion tests were conducted with these organic compounds to demonstrate the advantage of the chromatographic assessment. Further, we conducted comparison experiments using a pyrene-functionalized column and other CNT-loaded columns to elucidate the characteristics of each CNT column. The chromatographic approaches using CNT columns would be beneficial for realizing CNT suspensions with improved CNT dispersibility.

## 1. Introduction

The affinity between carbon nanotubes (CNTs) and organic molecules is of crucial importance because CNTs are prone to agglomerate in any media without the aid of dispersant (e.g., surfactant) [1]. Insufficiently dispersed CNTs do not display their superior properties such as high electrical conductivity, thermal conductivity, and so on. To fabricate composite materials exploiting the full potentials of CNTs, for instance, requires efficient exfoliation of CNTs [2,3]. Basically, media possessing high affinity to CNTs can disperse them more stably than media with low affinity. Determining the affinity between them can lead to a rational design for experimental systems.

Several methods for determining affinities between CNTs and organic compounds have been developed so far. One of the representative methods for determining the affinity is based on solubility parameters. The well-known Hildebrand solubility parameter has been widely used for estimating the solubility of polymers in specific solvents. The Hildebrand solubility parameters have been modified to Hansen solubility parameters (HSPs), composed of three parameters (*δ*_D_: dispersion term; *δ*_P_: dipolar term; *δ*_H_: hydrogen bonding term) divided from the Hildebrand ones [4]. In addition to the prediction of polymer solubility, Hansen solubility parameters have been widely investigated in terms of dispersion stability of nanomaterials not limited to spherical inorganic nanoparticles [5], but including nanocarbons such as CNTs [6], graphene [7], graphene oxide [8], nano-onion [9], and so on. In particular, CNTs are principally not dispersible in most solvents, and thus the estimation of solubility parameters for them possesses practical importance to survey the dispersibility to diverse kinds of solvents. Determining the HSPs of CNTs, however, requires a lot of experimental data on CNT dispersions using diverse kinds of solvents with different HSPs, which takes a lot of effort. In addition, the methodology to determine the degree of dispersibility, which is required to calculate HSPs, relies on the discretion of individual researchers and lacks consensus [10,11]. Although HSPs have advantages in predicting the affinity (dispersibility) not only for single solvents but also for solutions composed of multiple solvents, the methodology could be problematic when dealing with diverse kinds of CNTs.

Another potential method to determine the affinity between CNT and organic compounds is exploiting CNTs as a stationary phase for column chromatography. In the initial stage of the research, column chromatography using CNTs as a stationary phase has been investigated to utilize the high specific surface area of CNTs [12]. CNTs intrinsically possess high surface areas and thus the interaction between CNT surface and organic molecules is expected to effectively affect the retention of these molecules. Immobilization of CNTs has been made by methods such as direct growth on column beads [13], covalent attachment via chemical modifications [14,15,16], and so on. Organic compounds such as small polycyclic aromatic hydrocarbons [15] can be separated due to the strong interactions such as π-π interactions between those compounds and CNT surfaces. After that, several studies have investigated the applicability of chromatography using noncovalently loaded CNT columns to quantitatively determine the affinities of organic compounds [17,18]. Retention of organic molecules on columns originates from the interaction between the CNT surface and molecules, and thus strong retention implies their strong interaction, namely strong affinity. These techniques have the potential to realize the facile and rapid determination of the affinity of organic compounds, which could aid in the selection of appropriate dispersing media for CNTs. However, the number of research studies concerning this characterization method is limited and thus the actual influence on dispersibility in selected solvents is still not clear.

In this work, we have fabricated liquid chromatography columns containing single-walled CNTs (SWCNTs) with a relatively large diameter (so-called supergrowth SWCNT) deposited on the amine-functionalized silica beads according to the literature [17,18] and characterized the affinity between CNTs and organic molecules. While previous research investigated the affinity of SWCNTs (HiPco CNTs) for the molecular design of CNT solubilizers [17], and to understand the interaction between protein and CNTs [18], our study has focused on the relationship between the chromatography-based affinity of organic compounds and its effect on the dispersibility of CNTs. Further, we have characterized HSPs as another evaluation methodology to quantify the affinity of CNTs and compared it to that quantified by the CNT column chromatography to extract the difference between these techniques. The comparison experiment using a pyrene-functionalized column and other CNT (oxidized SWCNT and multi-walled CNT (MWCNT)) columns was also conducted to elucidate the characteristics of these CNT columns.

## 2. Materials and Methods

### 2.1. Materials

In most parts of this manuscript, single-walled CNTs (ZEONANO^®^ SG101, commercially available CNTs fabricated by the supergrowth procedure [19], produced by Zeon corporation, Tokyo, Japan) were used as a loaded material for column applications. For comparison, oxidized supergrowth SWCNT (fabricated by heating to 500 °C with a ramp rate at 1 °C/min under an air flow of 2 L/min) and MWCNT Knanos-400T (Kumho Petrochemical Co., Ltd., Seoul, Republic of Korea) were also used. Amine-functionalized silica beads CHEMCOBOND 3.5NH2 were purchased from Chemco Plus Scientific Co., Ltd., Osaka, Japan. *N*-methylpyrrolidone (NMP), methanol, toluene, *p*-xylene (*p*-Xy), 1,2,4-trimethylbenzene (1,2,4-TMB), *p*-chlorotoluene (*p*-CT), *o*-chlorotoluene (*o*-CT), *p*-bromotoluene (*p*-BT), *N*,*N*-dimethylacetamide, methyl ethyl ketone, acetophenone, chloroform, diethylene glycol, hexane, phenyl benzoate, naphthalene, 1-methylnaphthalene, and 2-methylnaphthalene were purchased from FUJIFILM Wako Pure Chemical, Osaka, Japan. All organic reagents were used without further purification. A pyrene-functionalized silica column (COSMOSIL 5PYE: pyrenylethyl groups are bonded to the surface) was purchased from Nakalai Tesque, Kyoto, Japan.

### 2.2. Fabrication Procedure of CNT Columns

**Figure 1:**
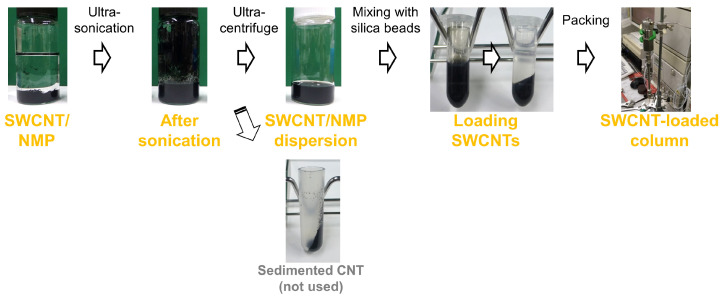
Photographic images of the preparation procedure of SWCNT columns.

Figure 1 shows the photographs of each step of the fabrication procedure for SWCNT columns used in this study, designed by referring to the literature [17,18]. Details of the procedure were as follows: first, SWCNTs were dispersed in NMP by a pre-treatment using an ultrasonic processor Nanoruptor NR-350 (Cosmo Bio Co., Ltd., Tokyo, Japan) for 1 min in a high-power mode and following intensive dispersion using an ultrasonic homogenizer Sonifier 250D Advanced (Branson, Brookfield, CO, USA) with 30% amplitude for 1 h under stirring. After the ultrasonication, obtained CNT dispersions were centrifuged by an ultracentrifuge Micro-ultracentrifuge GXII (Hitachi-koki, Tokyo, Japan) at 57,000 rpm (210,000× *g*) for 10 min, followed by extraction of supernatant (upper 3.5 mL from ca. 5 mL of dispersion and sediment) to be used for CNT loading. The CNT concentration of the obtained supernatant was adjusted to 0.025 mg/mL by adding an appropriate amount of NMP via referring to the calibration curves determined from UV-vis spectra obtained by using a UV-vis spectrometer UV-3600 (Shimadzu, Kyoto, Japan). Then, 20 mL of the diluted supernatant was mixed with amine-functionalized silica beads (0.50 g), and the mixture was rotated for 1 h using a rotator RT-5 (TAITEC, Saitama, Japan). Obtained CNT-loaded silica beads were packed into a rod as follows: 20 mL of the suspension was centrifuged at 3000 rpm (1650× *g*) for 3 min using a centrifuge H-36α (KOKUSAN, Saitama, Japan), and then 16 mL of the supernatant was replaced with chloroform/diethylene glycol (4:1 *v*/*v*). These centrifugation and supernatant exchange processes were repeated three times. Finally, 4 mL of the slurry was collected and then packed in a 2.1 mm i.d. × 125 mm empty column (Chemco Plus Scientific Co., Ltd.) by flowing chloroform/hexane (1:1 *v*/*v*) at 20 MPa. In this paper, the term “SWCNT column” indicates the column loading pristine supergrowth SWCNTs.

The fabrication of columns containing other kinds of CNTs was carried out using the same procedure as described above. For MWCNTs, 50 mL of 0.05 mg/mL CNT supernatant was mixed with the silica beads.

### 2.3. Chromatographic Analysis

Fabricated CNT columns have been characterized by using a gel permeation chromatography system HLC-8320GPC EcoSEC (Tosoh Bioscience, Tokyo, Japan) operating as a liquid chromatography system. The eluent was 50/50 (*v*/*v*) water/methanol solution at 40 °C, and the flow rate was fixed to 0.2 mL/min. Analytes dissolved in methanol were injected and detected by UV light at the wavelength of 220 nm. Blank measurement was conducted without dissolving analytes. The values of retention times were adopted from the peak top of the chromatograms and adjusted retention times were calculated by subtracting holdup time determined by blank measurement from retention time.

### 2.4. Evaluation of Dispersibility of CNTs

Evaluation of dispersibility of CNT was conducted by dispersing CNTs by stirring and measuring the height of CNT agglomerates after dispersion: CNTs (20 mg) were dried under vacuum for 3 h at 180 °C, and then 40 mL of dispersing solvents were added to them, and the resulting mixtures were stirred by a magnetic stirrer for 24 h at 1000 rpm. The degree of dispersion was determined by dividing the height of the CNT agglomerations observed after 2 h from the dispersion process by the height of the dispersing solvent.

### 2.5. Other Characterizations

Optical microscope images were obtained with a digital microscope VHX-7000 (Keyence, Osaka, Japan). HSPs of organic compounds for the calculation of HSP distance values were adopted from a database in software for Hansen solubility parameter HSPiP (version 5.3.05).

## 3. Results and Discussion

**Figure 2:**
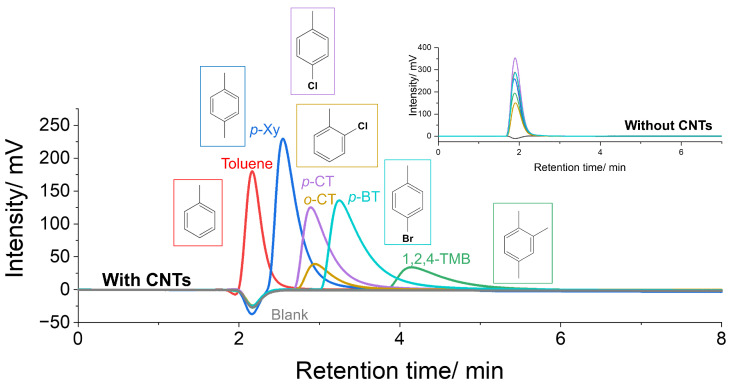
Liquid column chromatograms of several kinds of aromatic compounds using a SWCNT column. (inset) Liquid column chromatograms of several kinds of aromatic compounds using an amine-modified silica column without SWCNTs.

Figure 2 shows the chromatograms of several kinds of aromatic compounds for a SWCNT column. A vertical axis of Figure 2 indicates the degree of detected absorption of UV light, closely related to the amount of analyte. Obviously, the retention times of all compounds differ from each other, indicating that each organic molecule interacts with the CNT surface to varying degrees. Toluene was detected at almost the same retention time as the blank, indicating almost no interaction with the CNT surface. Halogen-substituted compounds resulted in longer retention times than toluene, and Br substitution (for *p*-BT) had a stronger effect on the affinity than Cl substitution (*p*-CT and *o*-CT). These results are presumably derived from halogen–π interactions, which are reported in a paper investigating the interaction between halogen-substituted benzene and polycyclic aromatic hydrocarbons or fullerenes [20]. Further, while constitutional isomers (*p*-CT and *o*-CT) were detected at almost identical retention times, retention times of a di-substituted compound (*p*-Xy) and tri-substituted compound (1,2,4-TMB) were quite different. A similar tendency has been observed for porous graphitic carbons [21], and analysis showed that an increasing number of methyl substituents resulted in more negative changes in molar enthalpy on adsorption. These results indicate that the affinity to the CNT surface can be significantly varied by the number of substitution groups even when the polarity of the molecule is virtually not changed. In contrast, the chromatograms shown in the inset of Figure 2 exhibit almost the same retention time for all compounds. These results indicate that while the CNT surface can interact with aromatic compounds and the degree of interaction between them differs from each other, the amine-functionalized silica surface without CNTs possesses virtually no interaction with all investigated compounds. In this work, we immobilized supergrowth SWCNTs in the columns, which exhibit relatively low G/D ratios (low degrees of crystallinity). This structural feature will contribute to the diversity of the chemical environment on the CNT surface, such as the existence of structural defects, surface functional groups, etc., and presumably lead to different results from other SWCNTs. The difference in organic compound–CNT affinity derived from the chemical environments will be briefly discussed in the latter part of this article.

**Figure 3:**
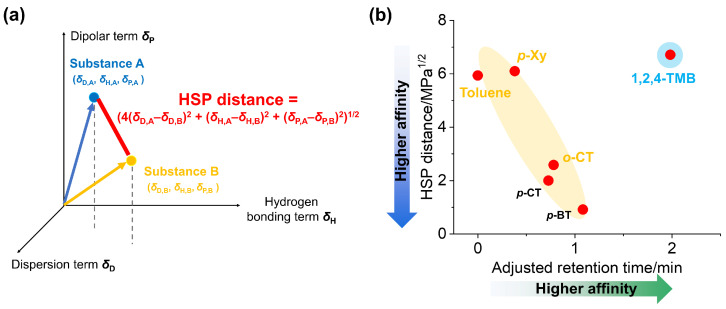
(**a**) Concept of the HSP distance in Hansen space. (**b**) Relationship between adjusted retention times obtained from SWCNT column and HSP distance values (from SWCNTs to organic compounds) of several kinds of aromatic compounds.

To compare the characterization of affinity with another analytical technique, we adopted the HSPs described in the Introduction. Figure 3a shows the concept of the HSP distance. As shown in Figure 3a, the HSP distance is essentially equal to the difference in the position in Hansen space, except for the factor of 4 on the dispersion term [4]. HSPs of used SWCNTs were adopted from the literature (dispersive term: *δ*_D_ = 19.4, polar term: *δ*_P_ = 6.0, hydrogen-bonding term: *δ*_H_ = 4.5) [22]. Basically, compounds with similar HSPs show high compatibility and, in many cases, high solubility or dispersibility among themselves. Thus, the above-defined HSP distance indicates the degree of incompatibility, and increasing HSP distance is prone to result in lower solubility (dispersibility).

Figure 3b shows the relationship between HSP distance values calculated from HSPs in a database and adjusted retention times obtained from SWCNT column chromatography for several aromatic compounds shown in Figure 2. Adjusted retention times were calculated by subtracting holdup time from retention times. It can be observed that most compounds shown in Figure 3b showed the trend in which decreasing HSP distance values lead to increasing adjusted retention times (highlighted in orange). As expected, HSP distance and (adjusted) retention times are closely related, and the compounds with similar HSPs to that of CNTs are estimated to exhibit long retention times. However, it can be clearly seen that 1,2,4-TMB displays a specifically longer retention time than expected from its high HSP distance value (highlighted in blue). These results indicate that (adjusted) retention times and HSP distance values do not necessarily exhibit similar tendencies for all compounds.

**Figure 4:**
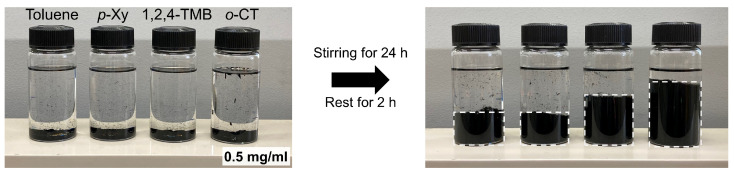
Appearance of CNTs in 4 kinds of solvents before and after the dispersion via vigorous stirring.

In general, solvents exhibiting a high affinity for materials are expected to show high dispersibility for them. To demonstrate the efficacy of the chromatographic analysis in searching CNT dispersants, we performed simple dispersion tests of CNTs using toluene, *p*-Xy, *o*-CT, and 1,2,4-TMB; the first three solvents exhibited a relationship between adjusted retention times and HSP distance values, while the last displays longer adjusted retention time than expected (Figure 3b). The degree of exfoliation was determined under the same dispersion conditions; specifically, we compared the percentage of the height of CNT agglomerates sufficiently after (more than 40 min) the dispersion process (stirring), where the sedimentation of CNT agglomerates reached the stable states. The properties of these solvents and the results of dispersion tests are listed in Table 1. It should be noted that while the degree of dispersibility of CNTs by stirring can be improved by using dispersant with high viscosity (typically > 50 mPa s) irrespective of molecular structure [23], these four kinds of solvents exhibit similar and relatively low viscosity (0.59 mPa s to 0.9 mPa s, shown in Table 1), and thus the effect derived from viscosity on the dispersibility could be negligible. The appearance of the obtained CNT dispersions by stirring is shown in Figure 4. The height of the CNT is relatively low for toluene and *p*-Xy (39% and 41%, respectively), indicating low CNT dispersibility, but it is higher for 1,2,4-TMB (59%). Note that the apparently high dispersibility in *o*-CT (80%) can be attributed to the high density of this solvent (Table 1), which provides buoyancy to CNTs, in addition to its low HSP distance value. The dispersibility tendency (1,2,4-TMB > toluene ≈ *p*-Xy) is consistent with the order of retention times but it cannot be explained by the order of HSP distance values (toluene ≈ *p*-Xy ≈ 1,2,4-TMB). The inconsistency in the HSP data is ascribable to the fact that HSPs are typically calculated from the partial contributions of functional groups [24], and thus HSP differences in polarity between compounds with a similar chemical structure (toluene, *p*-Xy, and 1,2,4-TMB) are basically small. Thus, the (adjusted) retention times can be useful for the estimation of CNT dispersibility using solvents with a similar chemical structure. Note that in the case of more intensive dispersion using ultrasonication resulted in different situations, as shown in Figure S1 and Table S1 in Supplementary Materials. In this dispersing condition, the highest CNT dispersibility can be obtained when 1,2,4-TMB is used as a solvent. Although further research is needed, the CNT column approach demonstrated in this paper has a high potential for estimating the dispersibility of CNTs, especially in the case of using solvents with similar chemical structures and low polarity.

**Figure 5:**
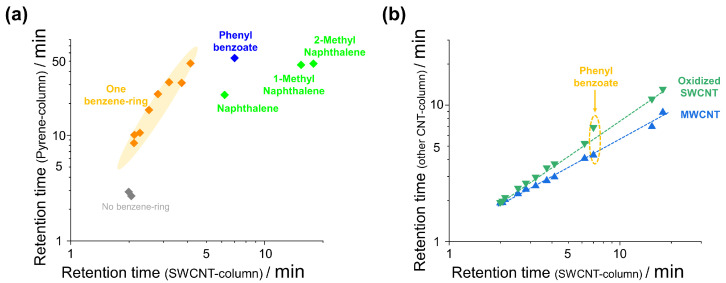
(**a**) Comparison of retention times of SWCNT column and pyrene-functionalized column. (**b**) Comparison of retention times of the SWCNT column to those of columns containing other kinds of CNTs.

Further, to highlight the characteristic properties of SWCNT columns used in this study, we performed comparison experiments using a pyrene-functionalized column and other CNT columns. Figure 5a displays the comparison of retention times of various organic compounds (mainly including one-benzene-ring aromatic compounds) for a SWCNT column and a pyrene-functionalized column. The pyrene-functionalized column is commercially available and displays strong π-π interactions. The pyrene-functionalized column exhibited long retention times (i.e., strong affinity) for aromatic compounds. In particular, this column showed a clear correlation with that of the SWCNT column for one-benzene-ring aromatic compounds (highlighted by orange color) (Figure 5a). However, organic compounds possessing two benzene rings (phenyl benzoate, naphthalene, and 1- and 2-methylnaphthalene) exhibited behavior different from those of the other compounds. These two-benzene-ring aromatic compounds showed relatively higher affinity to the SWCNT column compared to the one-benzene-ring aromatic compounds. This difference presumably originated from the difference in the degree of the extension of π-conjugated systems. While pyrene is a relatively small polycyclic aromatic hydrocarbon, CNTs correspond to rolled graphene sheets with a large contact area; note that the contact area governs the affinity (aromaphilicity) of aromatic substances for CNT surfaces [25]. Thus, the interaction of two-benzene-ring aromatic compounds with pyrene is relatively weak compared to that with CNT surfaces. These results indicate that the pyrene-functionalized column is not applicable for estimating the affinity of compounds, especially those multiple benzene rings, for CNTs.

Moreover, we conducted comparison experiments on other two kinds of CNTs: oxidized SWCNTs and MWCNTs. Oxidized SWCNTs were fabricated by mild air oxidation, which can typically introduce carboxyl and carbonyl groups to the CNT surface [26]. The MWCNTs used to possess a relatively long length (ca. 100 μm according to the supplier). Columns with these CNTs were fabricated via the same procedure as that employed in the pristine SWCNT column preparation, except for adjusting the loading amount of MWCNTs. Liquid chromatography analysis on these columns was conducted under identical conditions to that used for the pristine SWCNT columns. The obtained retention times for several organic compounds on the columns were plotted as a function of those on the pristine SWCNT columns (Figure 5b). Apparently, the retention times on both columns, in particular the MWCNT column, exhibited a trend similar to that of the pristine SWCNT column. The MWCNTs used in this study show relatively low crystallinity and long length [27], and both structural features can also be seen in the characteristics of the supergrowth SWNCTs. The results suggest that CNT curvature has an insignificant effect on the retention of these aromatic compounds. The oxidized SWCNT column also exhibited a trend similar to that of the pristine one, but only phenyl benzoate (emphasized by an arrow in Figure 5b) displayed a slightly longer retention time than expected, which may be a sign of its interaction with polar functional groups introduced by the oxidation process.

## 4. Conclusions

We have successfully fabricated SWCNT columns containing supergrowth SWCNTs and characterized their affinity with organic compounds by using them as a stationary phase for liquid chromatography. The tendency of affinity between organic compounds and SWCNTs was estimated by the adjusted retention times and compared to those estimated from Hansen solubility parameter (HSP) distance values. It was found that most of the investigated organic compounds exhibited a similar tendency to that obtained from HSP distance values, but 1,2,4-TMB deviated from this correlation. Simple dispersing experiments elucidated that affinities determined from retention times of SWCNT columns are effective in estimating the dispersibility of CNTs in organic compounds. The comparison experiments, using a pyrene-functionalized column, an oxidized SWCNT column, and an MWCNT column, demonstrated that the pristine SWCNT column exhibited relatively longer retention times for compounds possessing two benzene rings, indicating the affinity of CNT for large aromatic compounds. Although the differences in affinity for aromatic compounds are minimal among the pristine SWCNT column and the other CNT columns, the oxidized CNT column exhibited a slight influence on the affinity of phenyl benzoate, implying the possibility of achieving desired affinity for specific compounds by tuning the CNT surface environment through surface modification techniques. The chromatographic approaches developed in this work exhibited the practicality of realizing CNT suspensions with good dispersibility. We believe that our methodology could be extended to other nanocarbon materials with the capability to be loaded to the columns (potentially, e.g., graphene oxide) for assessing their dispersibility in organic compounds.

## Figures and Tables

**Table 1:** Properties of dispersing solvents used in Figure 4 and their dispersibility for CNTs.

	Toluene	*p*-Xylene (*p*-Xy)	1,2,4-Trimethylbenzene (1,2,4-TMB)	*o*-Chlorotoluene (*o*-CT)
Viscosity/mPa s (20 °C)	0.59	0.6475	0.9	0.884 (30 °C)
Density/g cm^−3^	0.867	0.861	0.876	1.087
CNT height percentage after stirring ^1^/%	39	41	59	80

^1^ CNT concentration: 0.5 mg/mL.

## Data Availability

Data are contained within the article and Supplementary Materials.

## Supplementary Materials

The following supporting information can be downloaded at: https://www.mdpi.com/article/10.3390/nano14100824/s1, Figure S1: appearance of CNT dispersions for 4 kinds of solvent dispersed via ultrasonication; Table S1: the dispersibility of CNTs in 4 kinds of solvents dispersed via ultrasonication; Figure S2: scanning electron microscope (SEM) images of SWCNTs on Si substrates deposited by spin coating the ultrasonicated CNT dispersions of 1,2,4-trimethylbenzene (1,2,4-TMB) and *o*-chlorotoluene (*o*-CT).
