# Supplementary material for: Chromatographic Assessment of Organic Compounds Using Carbon Nanotubes: The Relationship between Affinity and Dispersibility
Source: Nanomaterials (Basel). 2024 May 8;14(10):824. doi: 10.3390/nano14100824 (PMC11124211; doi:10.3390/nano14100824)
Supplement: Supplementary file 1 [file nanomaterials-14-00824-s001.zip › nanomaterials-2990690-supplementary.pdf]

Supplementary Materials

Article

Chromatographic Assessment of Organic Compounds Using Carbon Nanotubes: The Relationship between Affinity and Dispersibility

Taiyo Shimizu [1], Ryoichi Kishi [1,*], Atsushi Hirano [2], Ken Kokubo [1] and Kenji Hata [1]

[1] Nano Carbon Device Research Center, National Institute of Advanced Industrial Science and Technology (AIST), AIST Tsukuba Central 5, 1-1-1 Higashi, Tsukuba 305-8565, Ibaraki, Japan

[2] Nanomaterials Research Institute, National Institute of Advanced Industrial Science and Technology (AIST), AIST Tsukuba Central 5, 1-1-1 Higashi, Tsukuba 305-8565, Ibaraki, Japan

* Correspondence: r-kishi@aist.go.jp

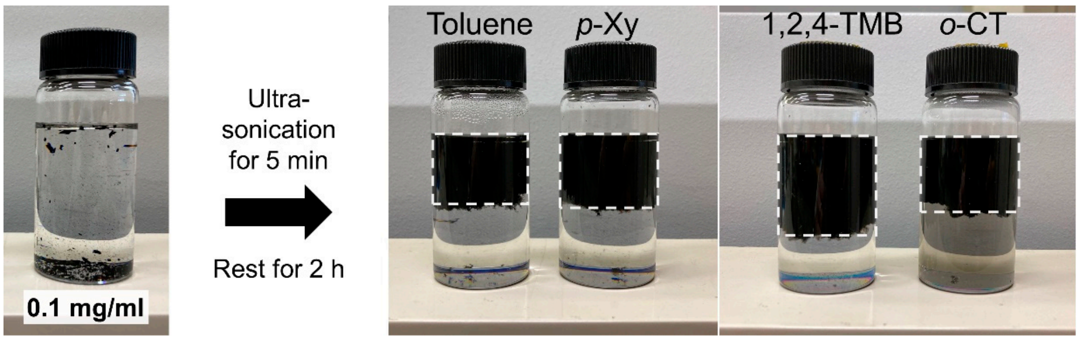

Figure S1 Appearance of SWCNT dispersions for 4 kinds of solvent dispersed via ultrasonication.

SWCNT dispersions via ultrasonication shown in Figure S1 were obtained by following procedure: SWCNTs of 4 mg were dried under vacuum for 3 h at 180 °C, and then dispersing solvent of 40 mL was added to them and dispersed by an ultrasonic homogenizer LUH300 (Yamato) at 60 % power for 5 min. The degree of dispersion was determined by dividing the height of the SWCNT agglomerations by the height of dispersing solvent after 2 h from the dispersion process.

Table S1 The dispersibility of SWCNTs in 4 kinds of solvents dispersed via ultrasonication.

	Toluene	*p*-Xylene (*p*-Xy)	1,2,4-trimethylbenzene (1,2,4-TMB)	*o*-chlorotoluene (*o*-CT)
CNT height percentage after ultrasonication [1]/ %	41	47	67	49

[1] CNT concentration: 0.1 mg/mL

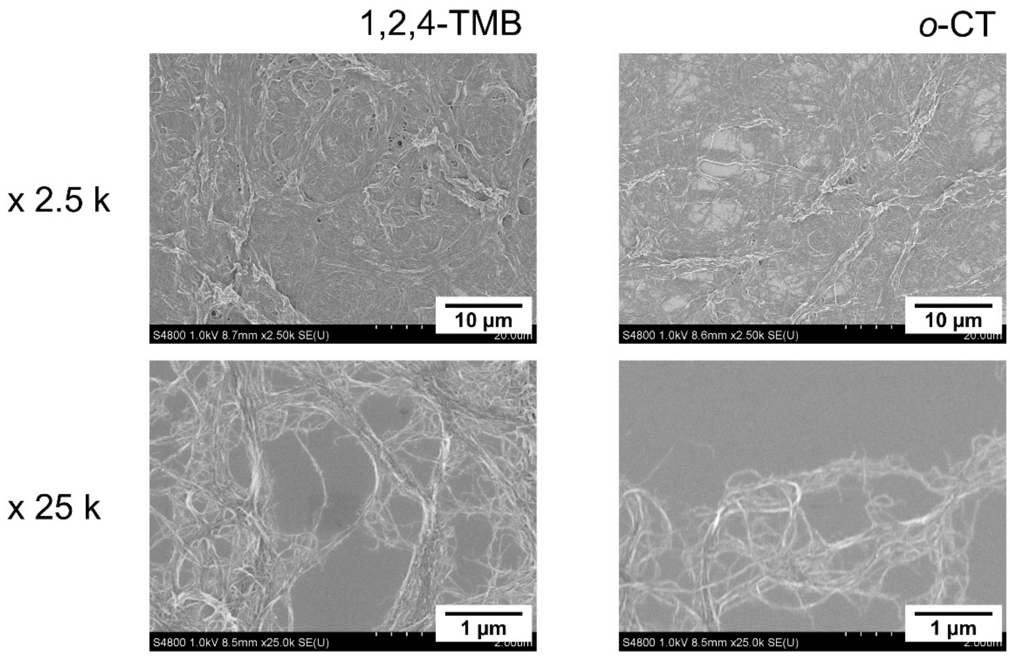

Figure S2 Scanning electron microscope (SEM) images of SWCNTs on Si substrates deposited by spincoating the ultrasonicated CNT dispersions of 1,2,4-trimethylbenzene (1,2,4-TMB) and *o*-chlorotoluene (*o*-CT).

We performed scanning electron microscopy (SEM) observation on the SWCNTs dispersed in 1,2,4-tirmethylbenzene (1,2,4-TMB) and *o*-chlorotoluene (*o*-CT) using a field emission scanning electron microscope S-4800 (Hitachi High-Tech). The SWCNTs were deposited on Si substrates by a spincoating process of SWCNT dispersions at 3,000 rpm for 30 s using a spincoater 1H-D7 (MIKASA). Obtained SEM images show no clear difference in morphologies such as bundle sizes, indicating the difficulty of the interpretation on the difference in dispersibility from nanoscale observations.
